# Feasibility and Implementation of the MINDY Tool in Routine Acute Psychiatric Inpatient Care

**DOI:** 10.1192/bjo.2026.11643

**Published:** 2026-06-30

**Authors:** Kishen Guruparan, Harith Ali, Alba Garcia-Melendez, Chania Lambrinudi, Kazuya Iwata

**Affiliations:** 1 Queen Mary University of London, London, United Kingdom; 2 East London NHS Foundation Trust, London, United Kingdom

## Abstract

**Aims:**

Structured assessment frameworks can enhance quality of clinical assessment and improve patient care, but must be feasible to deliver consistently in psychiatric settings. Acute inpatient wards face fluctuating acuity, staffing pressures and competing clinical priorities, which can limit the implementation of structured assessments. The Mental State Examination (MSE) remains central to psychiatric assessment but few tools have been evaluated for routine use in ward environments. MINDY is a structured mental state and risk assessment tool intended for routine inpatient use.

The aim was to evaluate the feasibility of implementing MINDY on an acute psychiatric ward. We hypothesised that MINDY could be delivered consistently during routine clinical practice with acceptable completion rates and minimal item-level missing data.

**Methods:**

This was a service-evaluation, retrospective and case-note review of clinical records on Opal Ward, Newham Centre for Mental Health - acute adult psychiatric inpatient service. Over a two-week period, three resident doctors completed MINDY assessments for patients under a single consultant team during clinical work, with one study day excluded due to staffing pressures. Implementation outcomes included rates of assessment completion, reasons for missed assessments and item-level completeness within submitted MINDY forms. Temporal trends across the study period and inter-individual variation were explored descriptively. All material was anonymised prior to analysis in accordance with NHS information-governance and General Data Protection Regulation (GDPR) policies. Analysis was performed by a fourth-year medical student, focusing on feasibility and implementation.

**Results:**

Of 66 expected assessments, 48 were completed (72.7%) across the two-week period. Reasons for 18 missed assessments included: patients on short-term leave (11/18; 61.1%), patient refusal (5/18; 27.8%) and lack of interpreter availability (2/18; 11.1%). Completion rates for MINDY assessments varied between individuals, ranging from 66.7% to 100%. No assessments were missed because of incomplete forms as item-level completeness within all completed MINDY assessments was 100%.

**Conclusion:**

In routine acute inpatient practice, MINDY demonstrated good feasibility. Nearly three-quarters of expected assessments were completed despite operational barriers, patient leave and refusal. Crucially, when assessments were undertaken, all core items were completed, indicating strong usability and low administrative burden for clinicians. These findings support MINDY as a deliverable ward-based tool for repeated use in inpatient environments. Future work should focus on strategies to reduce missed assessments and evaluating scalability across multiple wards and clinician groups. Once validated, MINDY could be delivered by the wider multidisciplinary team, improving feasibility.

